# Evaluation of suitable reference genes for gene expression studies in porcine PBMCs in response to LPS and LTA

**DOI:** 10.1186/1756-0500-6-56

**Published:** 2013-02-08

**Authors:** Mehmet Ulas Cinar, Mohammad Ariful Islam, Maren Pröll, Hakan Kocamis, Ernst Tholen, Dawit Tesfaye, Christian Looft, Karl Schellander, Muhammad Jasim Uddin

**Affiliations:** 1 Institute of Animal Sciences, Unit of Animal Breeding and Husbandry, University of Bonn, Endenicher Allee 15, 53115, Bonn, Germany; 2 Erciyes University, Faculty of Agriculture, Department of Animal Science, 38039, Kayseri, Turkey; 3 Department of Medicine, Faculty of Veterinary Science, Bangladesh Agricultural University, Mymensing, 2202, Bangladesh; 4 Department of Histology and Embryology, Veterinary Faculty, Kirikkale University, 71450, Kirikkale, Turkey

**Keywords:** Reference genes, PBMC, LPS, LTA, Pigs

## Abstract

**Background:**

As an *in vitro* model porcine peripheral blood mononuclear cells (PBMCs) is frequently used as for immunogenetic research with the stimulation of bacterial antigens. To investigate the immunocompetence of PBMCs for recognition of Gram-positive and Gram-negative bacteria and in order to dissect the pathogenesis of diseases, gene expression assay is most commonly used. The gene expressions are required to normalize for reference genes which have tremendous effect on the results of expression study. The reference genes should be stably expressed between different cells under a variety of experimental conditions, but recent influx of data showed that expression stability of reference genes are varied under different experimental conditions. But data regarding the expression stability of reference genes in porcine PBMCs are limited. Therefore, this study was aimed to know whether the expression stability of commonly used reference genes in PBMCs is affected by various bacterial antigens under different experimental conditions in pigs.

**Results:**

The mRNA expression stability of nine commonly used reference genes (B2M, BLM, GAPDH, HPRT1, PPIA, RPL4, SDHA, TBP and YWHAZ) was determined by RT-qPCR in PBMCs that were stimulated by LPS and LTA *in vitro* as well as cells un-stimulated control and non-cultured were also consider for this experiment. mRNA expression levels of all genes were found to be affected by the type of stimulation and duration of the stimulation (P < 0.05). geNorm software revealed that in case of irrespective of stimulation (without considering the type of stimulation), RPL4, PPIA and B2M were the most stable reference genes in PBMCs; in case of the control group, PPIA, BLM and GAPDH were the most stable reference genes. PPIA, B2M and RPL4 were the most stable reference genes in LPS stimulated PBMCs; and YWHAZ, RPL4 and PPIA were the most stably expressed reference genes in the case of LTA stimulated PBMCs. When LPS was used combined with LTA for the stimulation, YWHAZ, B2M and SDHA remained the most stable genes. PPIA, BLM and GAPDH were found to be most stably expressed reference genes when PBMCs were not cultured. NormFinder revealed different sets of stably expressed reference genes in PBMCs under different experimental conditions. Moreover, geNorm software suggested that the geometric mean of the three most stable genes would be the suitable combination for accurate normalization of gene expression study.

**Conclusion:**

There was discrepancy in the ranking order of reference genes obtained by different analysing algorithms (geNorm and NormFinder). In conclusion, the geometric mean of the RPL4, B2M and PPIA seemed to be the most appropriate combination of reference genes for accurate normalization of gene expression data in porcine PBMCs without knowing the type of bacterial pathogenic status of the animals and in the case of mixed infection with Gram-negative and Gram-positive bacteria. In case of PBMCs without any stimulation, PPIA, BLM and GAPDH could be suggested as suitable reference genes.

## Background

Peripheral blood mononuclear cells (PBMCs) consisting of lymphocytes and monocytes/macrophages are vital immune cells playing crucial roles in immune system. In response to the bacterial antigens, PBMCs produce different Toll-like receptors and cytokines lead to the immediate innate immune responses [1-3]. As an *in vitro* model, PBMCs stimulation with bacterial antigens is being frequently used for immunogenetic research in pigs [1,2,4,5]. Lipopolysaccharide (LPS) and lipoteichoic acid (LTA) are the pathogen associated molecular patterns (PAMPs) of the Gram-negative and the Gram-positive bacterial cell wall, respectively that cause activation of an inflammatory response *in vitro* as well as *in vivo*. Gene expression assay is a common way to investigate the defensive role of PBMCs in the bacterial infections as well as to dissect the pathogenesis of diseases. With this purposes, several studies focusing on gene expressions have been conducted in PBMCs *in vitro*[3,6-8]. The gene expression values are required to be normalized with suitable reference genes in order to avoid any false positive result in the expression study [9]. Therefore, it is crucial to know whether the expression stability of reference genes in PBMCs is affected by various PAMPs from infectious agents but these data are currently unavailable for pigs.

Quantitative real-time PCR (RT-qPCR) is a powerful technique for gene expression studies, which have become increasingly important in a large number of clinical and scientific fields [9,10]. Being fast, efficient, does not require post-PCR processing and functions over a large dynamic range of starting cDNA quantities makes it one of the most favourable mRNA quantification method. However, RT-qPCR can suffer from certain limitations which can lead to substantial variability in expression measures. One of the most important issues is to the selection of appropriate normalization factors to account for any errors and differences generated through the multi-step process involved in producing cDNA [11]. The most accepted approach for mRNA quantification is normalization of the expression level of a gene of interest (target gene) to the expression level of a stably expressed internal reference gene. The normalization adjusts for differences in the quality or quantity of template RNA or starting material and differences in RNA preparation and cDNA synthesis, since the reference gene is exposed to the same preparation steps as the gene of interest. This allows the direct comparison of normalized transcript expression levels between samples. The use of internal control genes (reference genes) assumes that their expression is invariant in the cells or tissue under study and with experimental treatments [12]. However, there is mounting evidence to suggest that the expression of internal reference genes may vary significantly under different experimental conditions opening the possibility that erroneous information [11]. As generally accepted that the selection of reference genes must be validated for a given tissue and set of conditions [13] and the use of multiple reference genes is viewed as a more robust, accurate and reliable approach to normalization [6,9,12,14]. Vandesompele et al. [9] suggested that geometric mean of multiple carefully selected reference genes is recommendable and suitable for accurate normalization.

Reference genes should ideally be constitutively expressed by all cell types and should not be affected by disease and experimental procedure. To date, a universal reference gene has not been identified. House keeping genes (HKGs) are most commonly used reference genes [9]. Although reference genes are expressed by any cell, their expression varies among different cell types/organs, age, sex and treatment or experimental conditions [6,15-21]. Use of HKGs as reference genes for a particular sample type should be, therefore, validated. However, the mRNA expression of reference genes from different cells and tissues [6,22-24] such as from AMs [15,21,25] may fluctuate due to the stimulation of infectious agents *in vitro*. Recently, we reported set of suitable reference genes from porcine AM cells [21]. PBMCs are being used as an important model to dissect the pathogenesis and genetics behind the infection through gene expression studies [1-3,7]. Therefore, the aim of the current study was to identify a set of stably expressed reference genes in porcine PBMCs irrespective of stimulation with PAMPs *in vitro* culture condition as well as in un-stimulated control and in non-culture condition.

## Methods

### Animals Blood Collection and Isolation of PBMC

At day 40, three German Landrace young pigs were bled by jugular vein to obtain blood samples for isolation of PBMC. The experiments were done according to the institutional guidelines and animal husbandry regulations of Germany [26]. Ten ml of blood from each pig were collected into a vacutainer tube containing anticoagulant (EDTA). Peripheral blood mononuclear cells from blood were isolated by gradient centrifugation using ficoll density gradient (Histopaque; Sigma-Aldrich, Munich, Germany) as describe earlier by Uddin et al. [3]. In brief, 10 ml of whole blood were carefully added on the top of 10 mL of Histopaque solution in a 50 ml conical tube. The tube was centrifuged at 400 × g for 30 min at room temperature. After centrifugation, the upper layer of the opaque interface containing mononuclear cells was aspirated and transferred to a new centrifuge tube. If the cells isolated were contaminated with red blood cells (RBC), they were treated with RBC lysis buffer (Invitrogen, Darmstadt, Germany) solution. After complete removal of RBC, the cells were washed twice with 10 mL of D-PBS (Invitrogen, Darmstadt, Germany) and centrifuged at 250 × g for 10 min at room temperature. The cells were then finally washed with Roswell Park Memorial Institute 1640 medium (RPMI-1640, Sigma-Aldrich, Munich, Germany), pelleted by centrifugation, and resuspended in RPMI-1640 media to make desired concentration of cells per millilitre.

### Stimulation of PBMCs with LPS and LTA

The PBMCs were plated in ultra-low attachment polystyrene 24-wells plate (CellStar, Frickenhausen, Germany) at 2 × 10^6^ cells in 1 ml medium in each well. The cells counting and concentration were adjusted by Haemocytometer (AbCam, Cambridge, UK). All plates were incubated at 37°C with 5% CO_2_ (Heraeus Instrument, Hanau, Germany) for 48 hours. After 1 hour incubation, adhered cells were stimulated with LPS of *Escherichia coli* 055:B5 (Sigma-Aldrich, Munich, Germany) (@10 μg per well), LTA of *Staphylococcus aureus* (Sigma-Aldrich, Munich, Germany) (@10 μg per well) and with a mixture of LPS and LTA (@10 μg or each per well) for 48 hours. The cells were then collected at 1, 4, 8, 12, 24 and 48 hours after stimulation for RNA extraction and stored at −80°C.

### RNA extraction and cDNA synthesis

Harvested PBMCs were washed in RPMI-1640 medium and the total RNA was extracted using Pico-Pure RNA isolation kit following the manufacturer’s protocol (Arcturus, Applied Biosystems, Darmstadt, Germany). Details of RNA isolation and cDNA synthesis were described in Cinar et al. (2012) [21]. Concentration of isolated RNA was determined spectrophotometrically by using the NanoDrop ND-8000 (Thermo Scientific, Braunschweig, Germany) instrument. Approximately 1.5 μg of total RNA for each sample was transcribed into cDNA by using SuperScript-II RT kit (Invitrogen, Darmstadt, Germany) for RT-qPCR analysis.

### Selection of reference genes

Nine reference genes (ACTB, GAPDH, HPRT1, B2M, SDHA, RPL4, YWHAZ, TBP and PPIA) were selected from Cinar et al. [21] which was done for stability of reference genes in stimulated alveolar macrophages. Details of primers and average Cq of stimulation groups are given in Table 1.

**Table 1:** Selected candidate reference genes, primers, and PCR reactions efficiencies

**Gene name**	**GenBank accession number**	**Primer sequence (5' → 3')**	**Amplicon length (bp)**	**Amplification efficiency (%)**	^**a**^**R**^**2**^	**Average Cq of cDNA (n = 2)**				
						**No culture**	**Control**	**LPS**	**LTA**	**Combined**
*B2M*	NM_213978	F:ACTTTTCACACCGCTCCAGT R:CGGATGGAACCCAGATACAT	180	89.20	0.992	20.4 ± 0.09	22.0 ± 0.63	32.2 ± 1.41	31.7 ± 1.39	30.38 ± 1.02
*BLM*	NM_001123084	F:TCCTCACCTTCTGCATTTCC R:GTGGTGGCTGAGAATCCTGT	152	93.12	0.993	26.5 ± 0.33	27.2 ± 0.79	35.2 ± 0.60	35.0 ± 0.72	35.7 ± 1.05
*GAPDH*	AF017079	F:ACCCAGAAGACTGTGGATGG R:ACGCCTGCTTCACCACCTTC	247	89.45	0.994	20.9 ± 0.78	23.5 ± 0.99	33.7 ± 0.79	33.5 ± 2.33	33.3 ± 1.34
*HPRT1*	NM_001032376	F:AACCTTGCTTTCCTTGGTCA R:TCAAGGGCATAGCCTACCAC	150	91.88	0.997	24.7 ± 0.26	26.5 ± 1.01	34.8 ± 1.41	34.3 ± 0.82	34.0 ± 0.57
*PPIA*	NM_214353	F:CACAAACGGTTCCCAGTTTT R:TGTCCACAGTCAGCAATGGT	171	91.32	0.997	19.3 ± 0.19	20.4 ± 0.78	31.0 ± 1.46	31.2 ± 1.29	29.9 ± 0.78
*RPL4*	DQ845176	F:AGGAGGCTGTTCTGCTTCTG R:TCCAGGGATGTTTCTGAAGG	185	90.21	0.993	19.8 ± 0.47	22.0 ± 0.79	31.9 ± 1.65	32.3 ± 1.67	30.7 ± 1.27
*SDHA*	DQ178128	F:AGAGCCTCAAGTTCGGGAAG R:CAGGAGATCCAAGGCAAAAT	149	92.24	0.996	25.6 ± 0.18	27.4 ± 0.53	34.7 ± 0.80	34.7 ± 0.85	34.7 ± 0.91
*TBP*	DQ178129	F:ACGTTCGGTTTAGGTTGCAG R:GCAGCACAGTACGAGCAACT	118	99.60	0.997	23.8 ± 0.13	24.7 ± 0.66	33.7 ± 2.61	32.4 ± 2.68	32.2 ± 0.45
*YWHAZ*	DQ178130	F:ATTGGGTCTGGCCCTTAACT R:GCGTGCTGTCTTTGTATGACTC	146	94.52	0.994	21.9 ± 0.25	23.9 ± 1.62	32.0 ± 1.29	32.7 ± 1.55	31.4 ± 0.94

### Quantitative real-time PCR (RT-qPCR)

**Figure 1:**
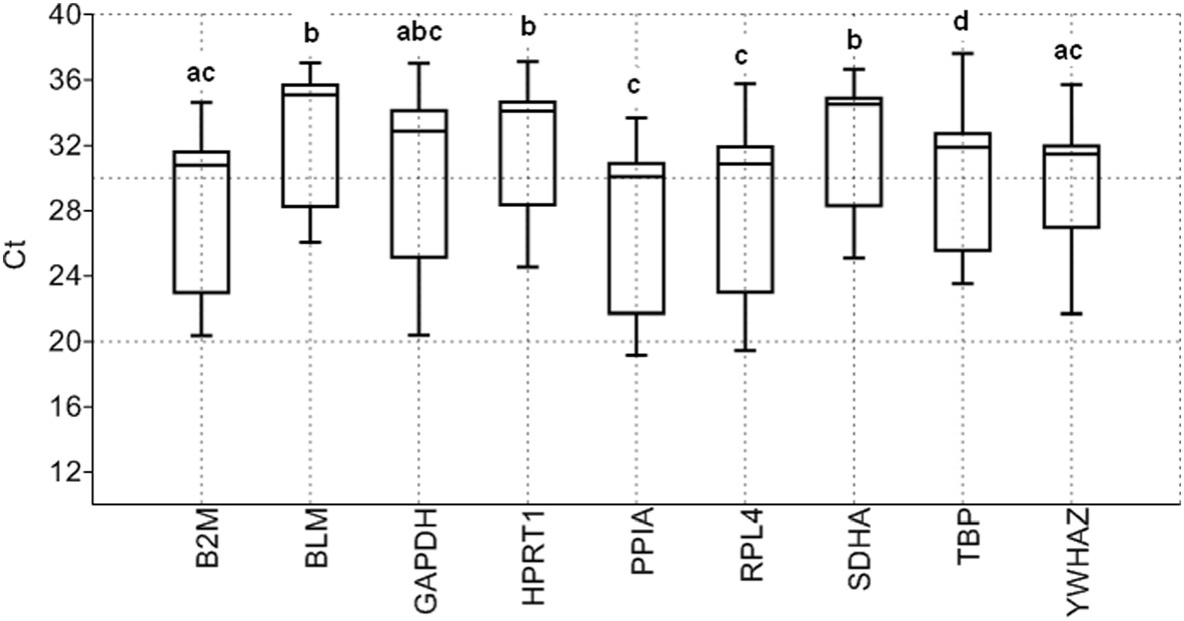
**Confirmation of amplicon size and primer specificity of studied genes. a**) Agarose gel electrophoresis showing specific reverse transcription PCR products of the expected size for each gene, M represents DNA size marker. **b-j**) Melting curve analysis for all amplicons.

Quantitative real-time PCR analysis was performed according to Cinar et al. [21]. Briefly, nine-fold serial dilution of plasmids DNA were prepared and used as template for the generation of the standard curve. Experiments were performed using the StepOnePlus™ Real-Time PCR System (Applied Biosystems, Darmstadt, Germany). Melting curve analysis was performed to verify the presence of gene-specific peaks and the absence of primer dimer (Figure 1b-j). Agarose gel electrophoresis was performed to test for the specificity of the amplicons (Figure 1a). To ensure repeatability of the experiments, all the reactions were executed in duplicate and the mean was used for further analysis.

### Determination of reference gene expression stability

The raw RT-qPCR amplification data was exported from the StepOne® software (Applied Biosystem, Darmstadt, Germany) to Microsoft® Excel. The averages of the Cq-values for each duplicate were used for stability comparison of candidate reference genes in the NormFinder and geNorm software. For easy understanding, the samples were grouped into 5 different categories such as LPS stimulated, LTA stimulated, LPS + LTA (combined), control and irrespective to stimulation group (when all the stimulated and non-stimulated control were considered together). The effect of stimulation and time on the expression of housekeeping genes was tested using GLM procedure of the SAS software (ver.9.2; SAS, SAS Institute Inc., Cary, NC, USA). Differences in gene expression levels between time and stimulation were determined using t-test in SAS software. *P* < 0.05 was considered statistically significant.

Cq-values of all samples were exported to Excel, ordered for use in geNormPlus software (15 days free trial version qBasePlus; http://www.biogazelle.com) and geNorm transformed to relative quantities using the gene-specific PCR amplification efficiency [27]. These relative quantities were then exported to geNormPlus to analyze gene expression stability [9]. The approach of reference gene selection implemented in geNorm relies on the principle that the expression ratio of two ideal reference genes should be identical in all samples, independent of the treatment, condition, or tissue type. Increasing variations in the expression ratio between two genes correspond to lower expression stability across samples. geNorm calculates the stability using a pairwise comparison model [9]. geNorm determines the level of pairwise variation for each reference gene with all other reference genes as the standard deviation of the logarithmically transformed expression ratios. In this way, the reference gene expression stability measure (*M* value) was calculated as the average pairwise variation of a particular gene with all other control genes included in the analysis [9,28]. Lower *M* values represent higher expression stabilities. Sequential elimination of the least stable gene (highest *M* value) generates a ranking of genes according to their *M* values and results in the identification of the genes with the most stable expression in the samples under analysis. geNorm was also used to estimate the normalization factor (NF_*n*_) using *n* multiple reference genes, by calculating the geometric mean of the expression levels of the *n* best reference genes [9]. The optimisation of the number of reference genes starts with the inclusion of the two genes with the lowest *M* value, and continues by sequentially adding genes with increasing values of *M*. Thus, geNorm calculates the pairwise variation V_*n*_/V_*n*+1_ between two sequential normalization factors NF_*n*_ and NF_*n*+1_ containing an increasing number of reference genes [9]. A large variation means that the added gene has a significant effect on the normalization and should preferably be included for calculation of a reliable normalization factor. Ideally, extra reference genes are included until the variation V_*n*_/V_*n*+1_ drops below a given threshold. According to geNorm, if V_n/n+1_ < 0.15 the inclusion of an additional reference gene is not required and the recommended number of reference genes is given by *n*[9].

NormFinder uses an ANOVA-based model [29]. The software calculates a stability value for all candidate reference genes tested. The stability value is based on the combined estimate of intra- and inter-group expression variations of the genes studied [29]. For each gene, the average Cq value of each duplicate reaction was converted to relative quantity data as described for geNorm, to calculate the stability value with NormFinder program [29]. The NormFinder reference tool was applied to rank the candidate reference gene expression stability for all samples with no subgroup determination (irrespective to stimulation) as well as with stimulation (LPS, LTA, and both LPS and LTA) as subgroup. A low stability value, indicating a low combined intra- and inter-group variation, indicates high expression stability [29].

## Results

### Total RNA quality and verification of amplicons

The average RNA concentration after extraction using Pico Pure was 9.28 μg/μl ± 0.6 (μg/μl ± SD). The optical density (OD) ratio A260/A280 nm measured with a Nanodrop spectrophotometer was 1.97 ± 0.16 (OD A260/A280 ratio ± SD). The results of the averaged amplification efficiencies are shown in Table 1. The amplification efficiencies for the nine candidate reference genes ranged between 89.20% and 99.60%. The agarose gel electrophoresis (Figure 1a) and melting curve analysis (Figure 1b-j and Table 1) revealed that all primer pairs amplified a single PCR product with expected size. Furthermore, sequence analysis of cloned amplicons revealed that all sequenced amplified fragments were identical to sequences used for primer design from GenBank (data not shown).

### Expression profiles of candidate reference genes

**Figure 2:**
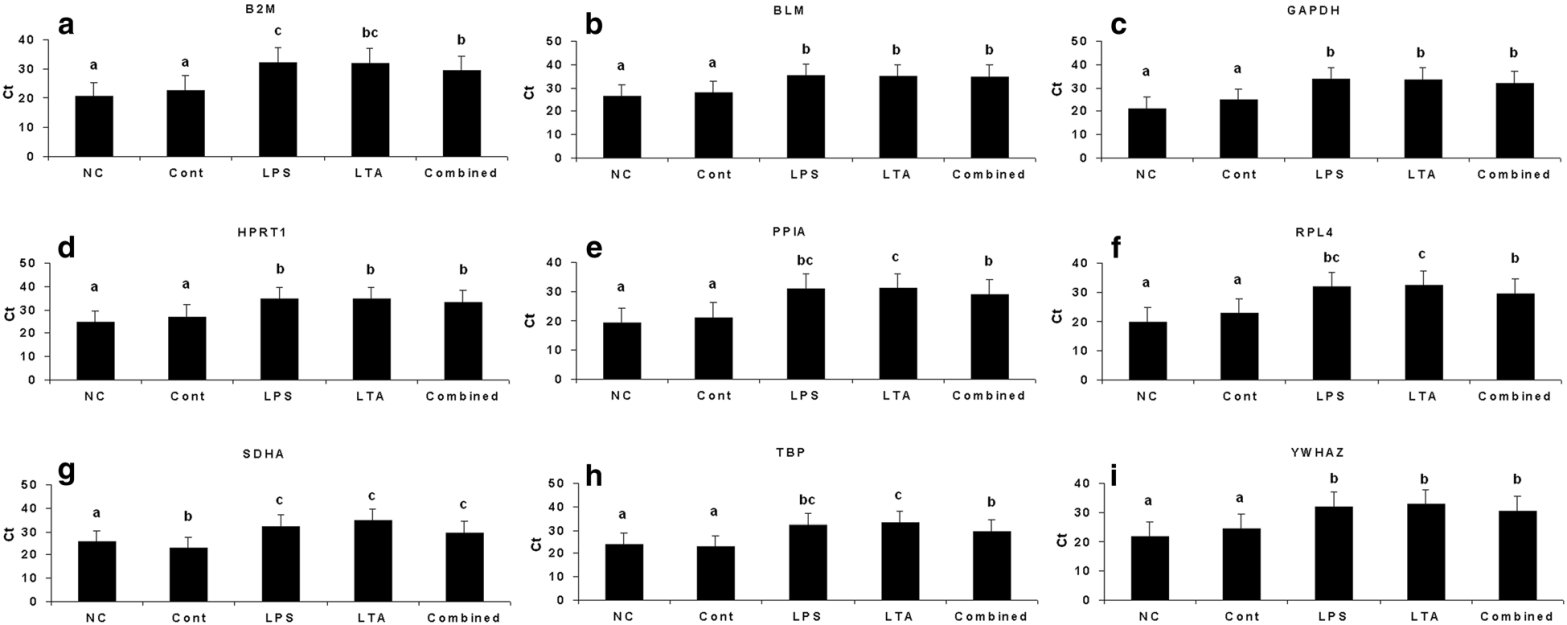
**Average cycle threshold (Cq) values of candidate reference genes tested in PBMCs under different conditions.** The values are the average RT-qPCR cycle threshold numbers (Cq values). The box plot indicates sample’s range, median, normality of the distribution, and skew of the distribution. Letters indicate a significant difference in average Cq value. Average Cq values that have the same letter are not significantly different (*P* > 0.05).

Transcript abundance of commonly used reference genes were analysed in the different samples by direct comparison of their cycle threshold (Cq), assuming equal Cq for equal transcript number since all RT-qPCR reactions were performed with an equal quantity of total RNA. Figure 2 showed that nine selected genes presented mean Cq-values that ranged from 27.78 to 33.04 cycles. Lowest and highest Cq-values were observed for *PPIA* (Cq 19.15) and *TBP* (Cq 37.62), respectively (Figure 2). Cq value of the selected genes showed a reasonable dispersion to moderately low expression levels (Figure 2). The highest variation was observed in GAPDH (min Cq 20.38 – max Cq 37.00) and followed by RPL4 (min Cq 19.44 – max Cq 35.74). BLM showed the lowest dispersion (min Cq 26.05 – max Cq 37.03) and mRNA expression as indicated by Cq-value around 33 cycles over the stimulations and culture conditions indicated by narrow whiskers of the box (Figure 2). SDHA was the second lowest varied (min Cq 25.09 – max Cq 36.65) and expressed gene (mean Cq 32.57) in our study (Figure 2). PPIA (mean Cq 27.78) was found to be highest expressed (mean Cq 27.78) gene among stimulations and different culture conditions (Figure 2). This followed by the expression of B2M gene (mean Cq 28.74). According to variance analysis, the expressions of eight genes were different from each other (*P* < 0.05) (Figure 2).

### LPS and LTA affect expression level of reference genes

**Figure 3:**
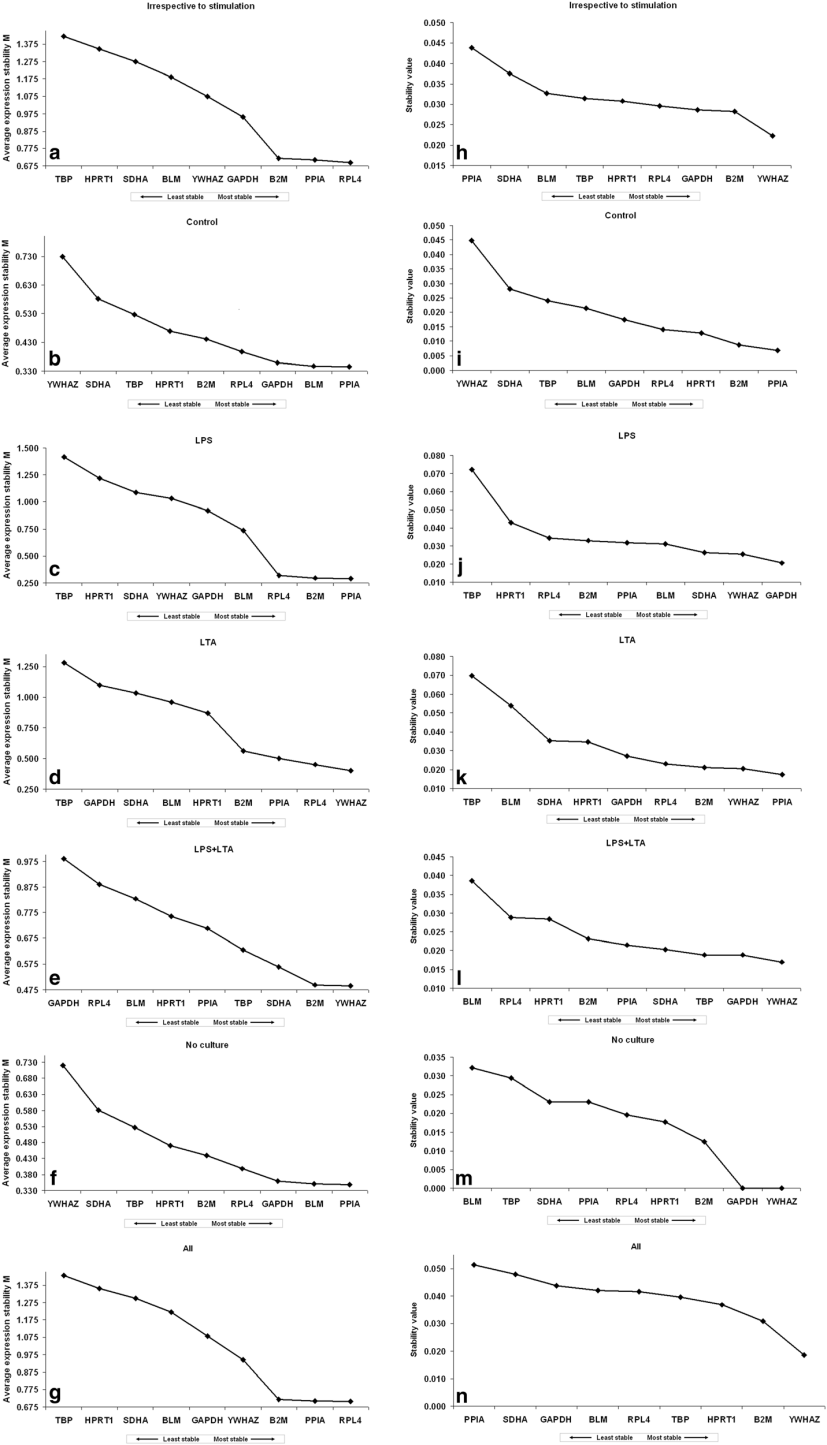
**Expression levels of a representative subset of nine reference genes. a**) B2M, **b**) BLM, **c**) GAPDH, **d**) HPRT1, **e**) PPIA, **f**) RPL4, **g**) SDHA, **h**) TBP and **i**) YWHAZ. NC (not-culture): cells which were not used for *in vitro* culture (in this case, PBMCs isolated from blood was used); Control: cells which were used for *in vitro* culture but was not stimulated; LPS: lipopolysaccharide; LTA: lipoteichoic acid; combined (LPS + LTA): lipopolysaccharide used together with lipoteichoic acid. Letters indicate a significant difference in average Cq value (*P* < 0.05).

PBMC mRNA expression differences were investigated in nine reference genes in no cultured, cultured with no stimulation, or stimulated with LPS, LTA or both. There were some fluctuations in the expression level of these genes in certain conditions. The expression differences of these genes are shown in Figure 3. The variance analysis results between treatment groups and time of stimuli to the PBMCs are shown in supplementary Table 1. Cell harvest time significantly affected the expression level of reference genes (*P* ≤ 0.02) (Supplementary Table 1). In all genes except SDHA, no statistical difference (*P* > 0.05) was observed between no culture (NC) and no stimulation (control) group (Figure 3). In the SDHA, no stimulation control group showed lower Cq value compared to no culture group (*P* < 0.05) (Figure 3g). When no culture group and no stimulation control group were compared with the stimulated groups, the expression levels of all genes were decreased in stimulated groups (Figure 3, Table 1). Within the stimulated groups, expression of BLM, GAPDH, HPRT1, SDHA, and YWHAZ was not effected from stimulation type (LPS, LTA or combined) (Figure 3). With LPS or LTA stimulation, mRNA expression levels of nine genes were decreased compared to control group (Figure 3a-i).

### Identification of optimal reference genes

**Figure 4:**
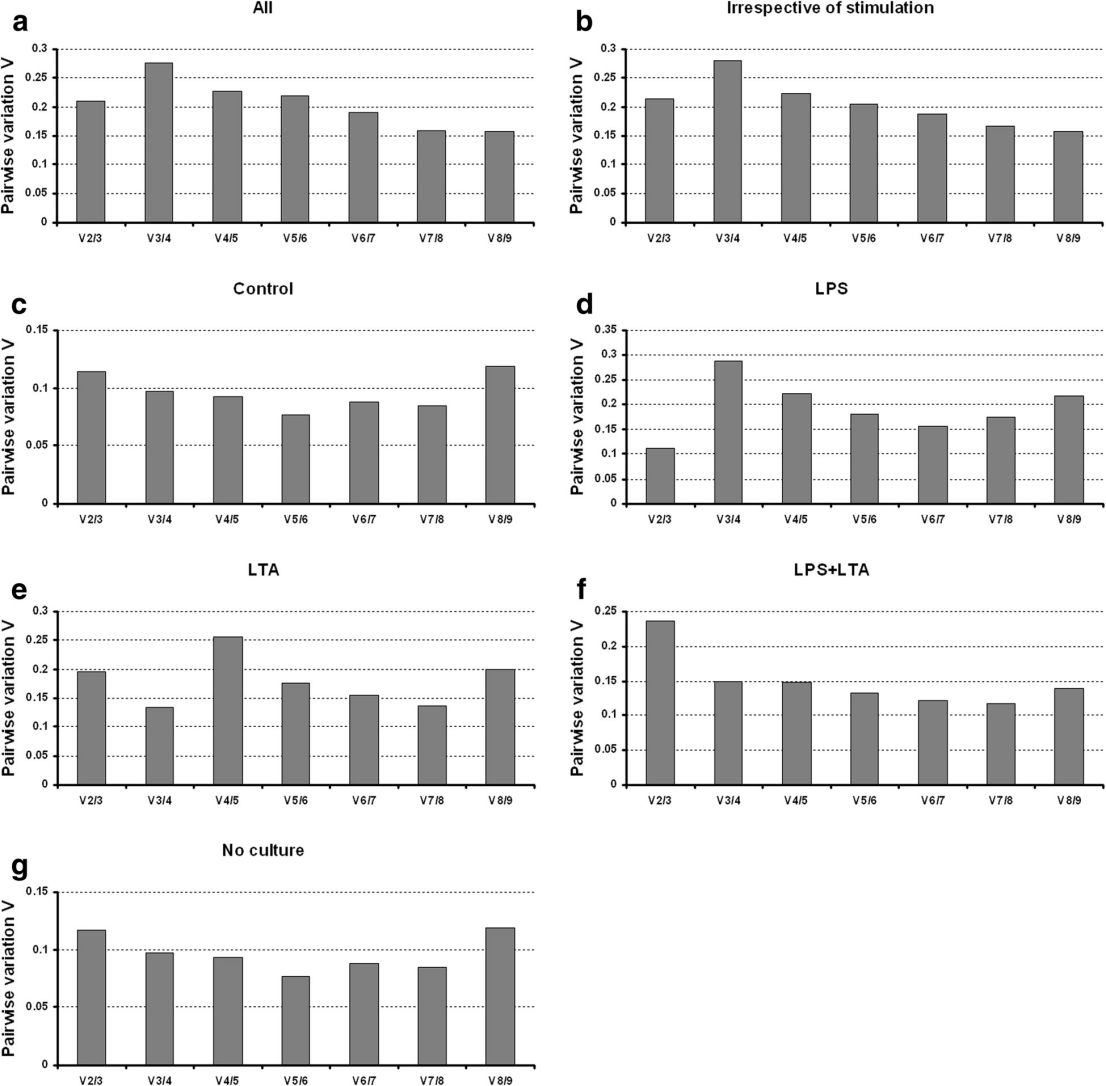
**Ranking of nine candidate reference genes using geNorm and NormFinder softwares. **(**a**-**g**) GeNorm ranks the candidate reference genes based on their stability parameter M. The lower the M value, the higher the expression stability. (h-n) NormFinder ranks the genes based on a calculated stability value. The lower the stability value, the higher the expression stability. All: when all types of stimulated + control + no-culture were considered together. Irrespective to stimulation: when all the stimulated and non-stimulated control groups were considered together; Control: no stimulation; NC (no culture): PBMCs did not culture, just after isolation from blood, it was used for RNA isolation in order to perform mRNA expression study. LPS: lipopolysaccharide; LTA: lipoteichoic acid; LPS + LTA (combined): lipopolysaccharide used together with lipoteichoic acid.

Transcription profiling using RT-qPCR assays was then performed with these nine candidate genes, in samples from the five different conditions of AM cultures (LPS, LTA, combined LPS and LTA, control and no culture). These raw Cq data were then analysed using different algorithms to identify the most suitable candidate genes. In each independent culture, the nine genes were ranked according to their gene expression stability measure “*M*” (Figure 4a-g, left panel) with using the geNorm algorithm. Stepwise exclusion of the least stable gene allowed the genes to be ranked according to their *M* value (the lower the *M* value, the higher the gene’s expression stability) [9]. The ranking of the candidate reference genes for all 5 different cases was shown in Figure 4a-g. According to *M* values, RPL4, PPIA and B2M were the most stable reference genes across the PBMCs based on their stability values without considering the type of stimulation of cells i.e. irrespective of stimulation group (Figure 4a). In case of the control group, geNorm showed that PPIA, BLM and GAPDH were the most stable reference genes (Figure 4b). When PBMCs were stimulated with Gram-negative bacterial product LPS, geNorm identified PPIA, B2M and RPL4 as the most stable reference genes (Figure 4c). YWHAZ, RPL4 and PPIA were the most stably expressed reference genes in the case of Gram-positive bacterial product (LTA) stimulation group (Figure 4d). When LPS was used combined with LTA for the stimulation of PBMCs, YWHAZ, B2M and SDHA remained the most stable genes (Figure 4e). Figure 4f shows the ranking of the nine candidate genes for PBMCs under no culture condition, where PPIA, BLM and GAPDH were found to be most stably expressed reference genes. Among all investigated groups, in four groups, TBP was found to be the least stable reference gene by geNorm (Figure 4a, 4c, 4d, 4g) except in control, LPS and LTA combined and in the no culture group; where YWHAZ was the least stably expressed gene in the control and no culture group (Figure 4b and 4f) and GAPDH was found to be the least stably expressed gene in the LPS and LTA combined group (Figure 4e).

NormFinder software ranked all reference genes according to their stability value (Figure 4h-n) [29]. Most of the genes or pairs of selected genes by two programs (geNorm and NormFinder) were not sufficiently stable among PBMCs under different conditions (Figure 4). In the irrespective to stimulation group, YWHAZ, B2M and GAPDH were found to be most stably expressed reference genes. (Figure 4h). In the non-stimulated control group PPIA, B2M and HPRT1 were the most stable reference genes (Figure 4i). In the LPS stimulated, LPS and LTA combined and no culture groups, GAPDH and YWHAZ were remained the most stable genes (Figure 4j, 4l and 4m). In the LTA stimulated group, PPIA, YWHAZ and B2M were ranked as the most stable reference genes (Figure 4k). The least stable reference genes which are detected by using NormFinder varied according to PBMC condition. For instance, in the control group YWHAZ was detected as least stable reference gene (Figure 4i), whereas for LPS stimulated and LTA stimulated groups TBP remained as the least stable reference gene (Figure 4j and 4k). In the LPS and LTA combined group and no culture group BLM was found to be least stable reference gene (Figure 4l and 4m).

### Determination of the optimal number of reference genes for normalization

**Figure 5:**
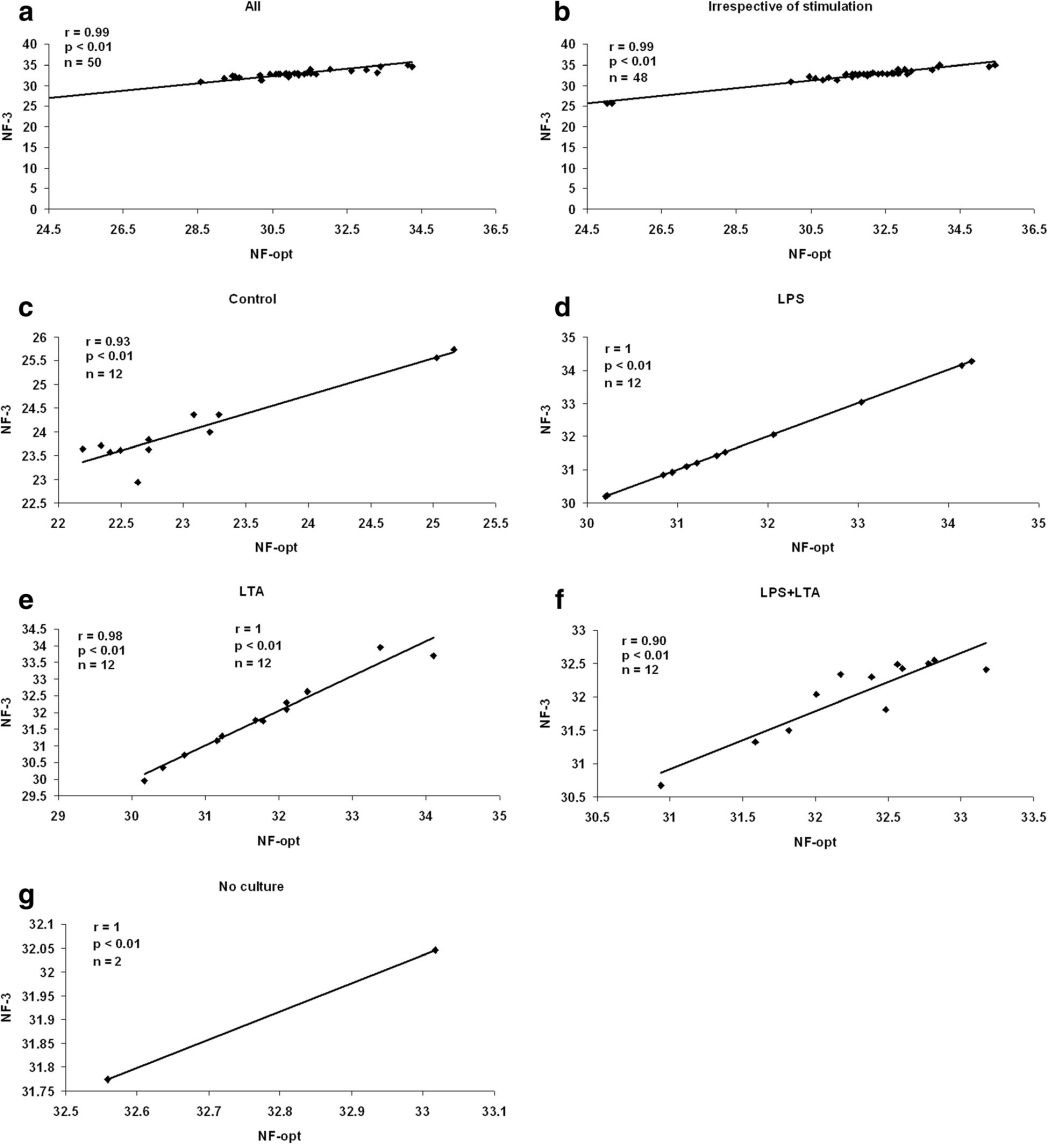
**Determination of the optimal number of reference genes for normalization.** The GeNorm software calculates the normalization factor from an increasing number of genes (starting with at least two) for which the variable V defines the pairwise variation between two sequential normalization factors. The lower the pairwise variation, the better is the combination of genes for reference. V8/9 for example (in Figure 5a ), shows the variation between the normalization factors of eight genes in relation to nine genes and shows that nine genes is the combination providing the lowest pairwise variation. For figure legends: see Figure 4.

**Figure 6:**
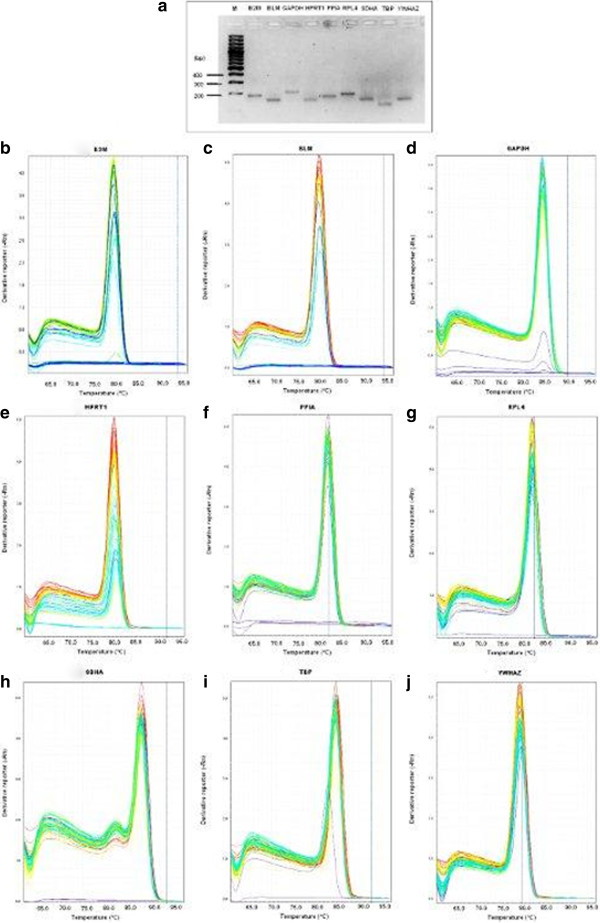
**Correlation between the NF of most three stable and optimal number endogenous control.** Pearson’s correlations between the NFs of three endogenous control genes (NF3) and optimal number of endogenous control genes (NFopt) for **a**) All (for explanation see Figure 4), **b**) irrespective of stimulation (for explanation see figure 4), **c**) non-stimulated control, **d**) LPS stimulated PBMCs, **e**) LTA stimulated PBMCs, **f**) LPS and LTA together used for stimulation, and **g**) PBMCs without culturing (for explanation see Figure 4).

The geNorm program calculates the normalization factor assessing the optimal number of reference genes for generating the *M* factor by calculating the pair-wise variation *V*. The pair-wise variation between these genes defines the variable *V*[9]. The lower the variable *V* is, the less variation. The overall results are shown in Figure 5. Eight endogenous reference genes were required for an accurate normalization factor in the all groups which represents cells *in vitro* cultured and non cultured together with LPS and/or LTA stimulation (Figure 5a). For the irrespective to stimulation group as shown in Figure 5b, nine endogenous control genes are necessary to obtain the lowest changing *V* values in the analysed samples. On the other hand, six endogenous reference genes were required for both control and no culture groups (Figure 5c and 5g). For the LPS stimulated group, three reference genes were required to obtain an accurate normalization factor (Figure 5d). For LTA stimulated group, combination four reference genes showed the lowest *V* value (Figure 5e). However, it is impractical to use excessive numbers of endogenous control genes for normalization, particularly when only a small number of target genes need to be studied or for rare samples that are very difficult to acquire [9,17,30]. Therefore, the use of the three most stable reference genes for the calculation of the NF was considered acceptable for the majority of experiments [9,17,30]. To verify that the use of three reference genes simultaneously is adequate for normalization of RT-qPCR data, the correlation of NF values between the geometric means of the three most stable genes and the optimal number of genes was calculated for each sample groups. As shown in Figure 6, there is a high correlation between the two NF measures (i.e., the theoretical optimal number and proposed number, three) for all groups including irrespective to stimulation group (*r* = 0.90 to 1, Pearson) (Figure 6a to g). This result demonstrates that the three most stable reference genes are sufficient for an accurate normalization of RT-qPCR data [9,17,30].

## Discussion

Normalization is a very important preliminary phase in the study of gene expression and requires the selection of suitable reference genes. Selection of reference genes that have a stable expression between the compared groups is crucial in gene expression studies and their expression variation in experimental treatments/ conditions in tissues or cells could lead to a misinterpretation of the results [9,31]. A number of studies have reported the validation of putative reference genes under different conditions [21,32,33]. However, the only study which is dealing with porcine PBMCs was performed by Facci et al. [34]. There they examined the expression stability of the 6 porcine genes in PBMCs with or without stimulation with lipolysaccharide (LPS) by using geNorm. Ideally, the internal control gene for quantitative gene expression studies should not be influenced by the conditions of the experiment. However, our study showed that expression of the reference genes was affected by *in vitro* culture conditions and stimulation type as well as interaction of stimulation type and duration (Figure 3; Additional file 1: Table S1). Similarly, in our previous study we showed that expression of nine selected reference genes were affected by stimulation type (LPS and/or LTA) and stimulation duration in case of porcine alveolar macrophages [21]. The expression stability of reference genes is reported to vary due to the treatment/disease conditions [15,18,35]. It has been shown that the expression of HKGs may be altered due to state of the organ [6,33], age [6,36,37] and experimental conditions [23-25,38]. Another consideration concerns the “Monte Carlo” effect, an inherent limitation of PCR amplification from small amounts of any complex template due to differences in amplification efficiency between individual templates in an amplifying cDNA population which may cause also expression differences [39]. Transcriptional expression differences of PBMCs due to LPS and LTA stimulation has been reported in pigs [1,3,34,40] and humans [41] but no study showed that these antigens single or together can affect the expression of reference genes in the porcine PBMCs.

Detecting inflammation based on gene expression signature, quantitative real-time PCR has became a powerful tool for the detection of inflammatory parameters, including cytokines and Toll-like receptors (TLRs) [3,40]. Commercial species-specific antibodies directed against pig cytokines and TLRs are not commonly available, therefore, RT-qPCR is particularly useful in pigs since. So, validation of reference genes has become a pre-requisite for reliable data normalization for gene expression studies and the inclusion of more than one reference gene is strongly recommended. Interestingly, guidelines for RT-qPCR gene expression analysis have been published to highlight the different variables involved and to show how to minimize their effect to improve accuracy and reproducibility [31]. So far, major attention has been focused on reliable reference genes to determine the profile of gene expression in pig organs [16,17,20,42-44]. There has not been a detailed study under different types of stimulation such as LPS, LTA and combined LPS and LTA that might be indicated Gram-negative, Gram-positive bacterial infection or co-infection of both types of bacteria. On the other hand, porcine polarized intestinal cells [44] and colonic explants [45] stimulated with a protozoan *Entamoeba histolytica in vitro* and stability of 7 and 9 reference genes were investigated, respectively. More closer, Facci et al. [34] studied expression stability of six reference genes *in vitro* LPS stimulated porcine PBMCs. In their experiment they examined the expression stability of the 6 porcine genes in PBMCs with or without stimulation with lipolysaccharide (LPS) by using geNorm. Stability of reference genes in porcine PBMCs and alveolar macrophages (AM) shows some similarities in LPS, LTA and LPS + LTA stimulation groups [21]. Although B2M found to be most stable gene in LPS stimulated AMs, it is identified as second most stable gene in LPS stimulated PBMCs. Both in PBMCs and AMs YWHAZ, PPIA and RPL4 were identified as the most stable reference genes. In the LPS + LTA combined group, two of the most stable three reference genes YWHAZ and SDHA were found to be common in PBMCs and AMs. Uddin et al. [6] found that mRNA expression of nine reference genes which were studied also in this experiment affected by age in un cultured PBMCs. However they did not test the stability ranking of reference genes according to tissues. Therefore, it is not easy to discuss stability ranking of our results with the result of Uddin et al. [6] in the post-weaned piglets. Because they used new born and two months old pigs in which our sampling falls in between these two age groups. There have been numerous research papers which have used single reference genes for normalisation of gene expression in PBMCs [1,46,47]. Therefore, we investigated expression stability of nine widely used reference genes in states of different inflammatory models. Xiang-Hong et al. [48] investigated expression stability of six potential reference genes in Bama miniature pig PBMCs under heat stress by using geNorm and NormFinder programs, whereas Uddin et al. [6] showed that the expression stability of nine reference genes vary in PBMCs collected from different ages pigs. Among all time points (day 1, 7, 14 and 21) RPL4 and TBP ranked as the two most stably expressed genes, except on day 21 when B2M was the most stable, whereas GAPDH were discarded as the least unstable [48]. Several studies has reported in differential reference gene expression in different porcine tissues such as fat- and muscle-type tissues [17,20], liver tissues [42], adipose tissues [36], stomach tissues [49], mesenchymal stem cells [50] and female reproductive organs [43]; however, since they did not investigate porcine PBMCs, it is difficult to compare our results with the literature findings.

The two approaches used to analyse the expression stability of the selected reference genes in our study were geNorm and NormFinder. There was not consistent agreement in the rank ordering, or the selection of the optimal candidates by different methods (Figure 4). No single reference gene was consistently identified as being the most stably expressed by geNorm or NormFinder. It is important to note that very similar discrepancies between these different algorithms have been observed in previous studies comparing statistical analysis methods [6,15,21,28,49,51]. The geNorm principle is based on the assumption that two ideal reference genes have identical expression ratios regardless of the conditions. The software thus provides the two genes that have the most similar expression profile throughout the samples [9]. NormFinder is more resistant to the presence of co-regulated genes, because it uses a different algorithm to establish the stability of the genes [29]. This software presents a stability value, which is directly related to the intra-group variance (when no subgroups are present) and is independent of the gene and sample [9]. It basically calculates which gene has the smallest variation over all samples [52]. Such discrepancy among different algorithms could be explained by genes’ co-regulation. Indeed, co-regulated genes may become highly ranked independently of their expression stabilities with geNorm software [29]. Moreover, NormFinder takes into account the variation across subgroups, thus avoiding artificial selection of co-regulated genes by analyzing the expression stability of candidate genes independently from each other [29]. However, limited number of porcine reference gene stability study [6,21] was performed with using different algorithms (e.g. NormFinder) other than geNorm [16,17,20,36,49].

## Conclusions

In conclusion, this investigation found evidence that there can be variation in the expression of commonly used reference genes in response to the stimulation with bacterial products or antigens. Due to the new influx of data suggesting alterations in mRNA expression according to bacteria type, we feel that beside therapy uses or experimental condition, there needs to be special consideration given to the selection of reference genes based upon the bacterial pathogen identification. This indicates that the choice of reference genes cannot be transposed from one study to the other without validation for the specifics of each experimental protocol. In general, we recommend using the geometric mean of RPL4, B2M and PPIA to guarantee suitable normalization in across the PBMCs with unknown pathogenic condition in pigs. Since in the most cases, Gram-negative and Gram-positive bacteria are observed together in porcine diseases, RPL4, B2M and PPIA might be an appropriate set of reference genes for the gene expression normalization in PBMCs studies. PPIA, BLM and GAPDH could be suggested in the case of PBMCs without any stimulation. This study offers an appropriate set of reference genes that might be used in the normalization of gene expression data *in vitro* cultured porcine PBMCs.

## Abbreviations

RT-qPCR: Quantitative real-time reverse transcriptase polymerase chain reaction; B2M: Beta-2-microglobulin; BLM: Bloom syndrome, Rect helicase-like; GAPDH: Glyceraldehyde 3-phosphate dehydrogenase; HPRT1: Hypoxanthine phosphoribosyltransferase 1; PPIA: Peptidylprolyl isomerase A (cyclophilin A); RPL4: Ribosomal protein L4; SDHA: Succinate dehydrogenase complex subunit A flavoprotein; TBP: TATA box binding protein; YWHAZ: Tyrosine 3/tryptophan 5-monooxygenase activation protein zeta polypeptide; NTC: No-template control; Cq: Cycle threshold; SD: Standard deviation; HKG: House keeping gene; PBMC: Peripheral blood mononuclear cell.

## Competing interests

The authors declare that they have no competing financial or other interest in relation to this work.

## Authors’ contributions

MUC performed the qPCR experiments, analysed data and prepared and edited the manuscript. MAI isolated PBMCs and made *in vitro* experiments. MJU analysed data and prepared the manuscript with MUC. DT, ET and CL edited the manuscript. KS criticized the experimental design and edited the manuscript. All authors read and approve the final manuscript.

## Supplementary Material

Additional file 1 **Relative expression of candidate genes and effect of treatment and time of stimuli on expression level.** Overall expression data of reference candidate genes. Summary of the Proc GLM (ver.9.2; SAS, SAS Institute Inc., Cary, NC, USA) analysis detecting effect of stimulation type, duration of stimulation *in vitro* and interaction on the mRNA expression of reference candidate genes. Click here for file
