# Supplementary material for: Evaluation of suitable reference genes for gene expression studies in porcine PBMCs in response to LPS and LTA
Source: BMC Res Notes. 2013 Feb 8;6:56. doi: 10.1186/1756-0500-6-56 (PMC3584940; doi:10.1186/1756-0500-6-56)
Supplement: Additional file 1 — Relative expression of candidate genes and effect of treatment and time of stimuli on expression level. Overall expression data of reference candidate genes. Summary of the Proc GLM (ver.9.2; SAS, SAS Institute Inc., Cary, NC, USA) analysis detecting effect of stimulation type, duration of stimulation in vitro and interaction on the mRNA expression of reference candidate genes. [file 1756-0500-6-56-S1.doc]

**Table S1 Relative expression of candidate genes and effect of treatment and time on expression level**

**(calculated by PROC GLM, SAS)**

|  | Mean±SD | Treatment | Time | Treatment*Time | R^2^ | Model |
| --- | --- | --- | --- | --- | --- | --- |
| B2M | 29.08±1.3 | <0.0001 | <0.0001 | 0.0089 | 0.990 | <0.0001 |
| BLM | 33.31±1.5 | <0.0001 | 0.02 | <0.0001 | 0.991 | <0.0001 |
| GAPDH | 31.02±2.0 | <0.0001 | 0.01 | 0.07 | 0.967 | <0.0001 |
| HPRT1 | 32.42±0.9 | <0.0001 | 0.008 | 0.007 | 0.980 | <0.0001 |
| PPIA | 28.13±1.0 | <0.0001 | <0.0001 | 0.0001 | 0.994 | <0.0001 |
| RPL4 | 29.23±1.1 | <0.0001 | <0.0001 | <0.0001 | 0.989 | <0.0001 |
| SDHA | 32.85±1.3 | <0.0001 | 0.01 | 0.03 | 0.980 | <0.0001 |
| TBP | 30.72±1.1 | <0.0001 | 0.02 | 0.01 | 0.936 | <0.0001 |
| YWHAZ | 29.99±0.9 | <0.0001 | <0.0001 | 0.001 | 0.989 | <0.0001 |
